# Mining is bad for health: a voyage of discovery

**DOI:** 10.1007/s10653-019-00367-7

**Published:** 2019-07-09

**Authors:** Alex G. Stewart

**Affiliations:** College of Life and Environmental Science, University of Exeter, Exeter, EX4 4RJ UK

**Keywords:** Pneumoconiosis, Economics, Determinants of disease, Prevention, Social support, Psychological stress

## Abstract

Mining continues to be a dangerous activity, whether large-scale industrial mining or small-scale artisanal mining. Not only are there accidents, but exposure to dust and toxins, along with stress from the working environment or managerial pressures, give rise to a range of diseases that affect miners. I look at mining and health from various personal perspectives: that of the ordinary man (much of life depends on mined elements in the house, car and phone); as a member of the Society for Environmental Geochemistry and Health (environmental contamination and degradation leads to ill health in nearby communities); as a public health doctor (mining health is affected by many factors, usually acting in a mix, ranging from individual inheritance—genetic makeup, sex, age; personal choices—diet, lifestyle; living conditions—employment, war; social support—family, local community; environmental conditions—education, work; to national and international constraints—trade, economy, natural world); as a volunteer (mining health costs are not restricted to miners or industry but borne by everyone who partakes of mining benefits—all of us); and as a lay preacher (the current global economy concentrates on profit at the expense of the health of miners). Partnership working by academics with communities, government and industry should develop evidence-based solutions. Employment, health, economic stability and environmental protection need not be mutually exclusive. We all need to act.

## Introduction

I have spent many years working at the interface between the environment, both natural and built, and health, in both general medical (family) practice and in community-focussed public health practice (e.g. Stewart 1990; Stewart et al. 2003; Herm et al. 2005; Stewart et al. 2010; Mahoney et al. 2015; Stewart and Hursthouse 2018). However, it was not until a colleague challenged me to contribute to a debate on mining and health that I seriously looked at the specific issues that link mining and health. The following is less of a regular review than a personal assessment of the ways mining affects health. I recount some of the considerations that made me pause for thought during and after the debate. I arrange them by various aspects of my life.

## Insight 1—as an ordinary man

First of all, I discovered that it is not possible to ignore the impact of mining on daily living. I own a house, a car and a mobile phone, and all are dependent on mining. In my house, various components arise from mining: the bricks come from clay while the attractive chimney breast is made from local slate. Nails and screws are either iron or zinc, while water pipes are made from copper, zinc, nickel and chrome. The window glass needs silica, feldspar and soda ash for its genesis, while the concrete base supporting the house consists of limestone, clay, shale and gypsum. Locks and hinges are also made from copper, zinc or iron, and the insulation is glass wool (silica, feldspar, soda ash) or expanded vermiculite.

Without the following mined essentials, the car would not function properly: glass (as above), battery (lead, zinc), paint (cadmium), steel (an alloy of iron and other metals) or aluminium, while under the bonnet there is a whole mix of elements, including nickel, copper, molybdenum, beryllium, vanadium, as well as a mineral (mica).

And my mobile phone contains gold coating the wires on the circuit boards (possibly the only gold I own), copper acting as a transistor in the circuit boards, tantalum to store electricity in the circuit boards, rare earth elements to provide colours and tungsten to help the phone vibrate.

To take only one example, tantalum is used by many industries and manufacturers in capacitors. It takes about one tonne of rock to produce 30 grams of tantalum. Most tantalum comes from Central Africa, much of it being panned from ore in the Democratic Republic of Congo by small-scale miners. The trade is under the control of militia groups who rule by murder, rape and brutality. Smuggling of the element is undertaken to avoid sanctions and tax, leading to exploitation and corruption (Bell 2014a, b). Not a healthy lifestyle.

And I learnt that small-scale artisanal mining has particular challenges that are not seen in large-scale mines, including the association of gold mining with health problems from psychosocial, cardiovascular, respiratory and sexual risks, nutritional, water and sanitation issues, and resulting in malaria, upper respiratory tract diseases, especially pulmonary tuberculosis and silicosis, and skin diseases, as well as the injuries and accidents more commonly associated with mining (Basu et al. 2015). Not a healthy occupation. Since artisanal mining may be the only source of income for these miners and families, it is important to find ways to improve their lot and make artisanal mining safer.

## Insight 2—a member of SEGH

As a medical doctor, I have found fellow members and the many meetings of the Society of Environmental Geochemistry and Health (http://www.segh.net/) which I have attended, along with this journal, very helpful over may years in exploring the links between the environment and health. My initial exploration on mining within the Society of Environmental Geochemistry and Health fold focussed on the environment, but soon led back to health.

Although mining provides resources that are essential to the basic needs of civilisation and the requirements of the high technology world that most of us live in, nevertheless, it can result in substantial environmental and human health problems. Across the world, mining contributes to erosion, sinkholes, deforestation, loss of biodiversity, significant use of water resources, dammed rivers and ponded waters, wastewater disposal issues, acid mine drainage and contamination of soil, ground and surface water, all of which can lead to health issues in local populations (Rajaee et al. 2015; CSIR 2013; Liao et al. 2016). Not a good reputation.

Underground coal mining is far more dangerous than surface mining, including the loathsome removal of whole mountaintops to access coal seams. One tonne of rock removal can produce a half tonne of coal. A much better return than mining for tantalum. However, between 10 and 21% of coal miners develop coal miners’ pneumoconiosis (black lung disease) from components in the dust (Blackley et al. 2018), while in China a prevalence of over 30% has been reported (Cui et al. 2015). An earlier systematic analysis of Chinese studies reported an overall prevalence of 6%, almost doubling to 11% in those with tuberculosis (Mo et al. 2013). Whatever the true rate of coal miners’ pneumoconiosis (the discrepancies might be due to different levels of exposure, different diagnostic criteria, different recording systems, different genetic susceptibilities or the like), it is too high as the disease is preventable. Classical silica-induced pneumoconiosis is found in older miners, both open-cast and underground, with a lengthy exposure (Leung et al. 2012) (see also below).

Globally, mining is a major source of particulate matter. Mining activities play an important but underappreciated role in the generation of contaminated atmospheric dust and aerosol and the transport of metal and metalloid contaminants (Csavina et al. 2012a; Meyer et al. 2015). Coarse particles form a large proportion of resulting dust particles and are usually too heavy to travel far, although they may still contribute to exposure of workers and nearby residents (Barbieri et al. 2014; Zota et al. 2016). The combination of mining activities and mechanical dispersion via water and wind has moved heavy metals around 4 km from a mine site in Iran (Mokhtari et al. 2018), while in Hunan Province, China, metal aerial contamination peaked around the 1 km mark. However, topography was more important than wind in the distribution of metals (Ding et al. 2017).

Fine particles (PM_10_ or smaller), such as those resulting from smelting operations or found in slag dumps or arising from the erosion of contaminated soil, disperse readily into the environment, often in association with aerosols, and may travel quite a lot further than anticipated (Reynolds et al. 2010; Meyer et al. 2015; Yu et al. 2019). Fine particles also penetrate more deeply throughout the respiratory system and are more likely to result in adverse health effects (https://www.esrl.noaa.gov/gmd/about/airquality.html; Csavina et al. 2012a, b; Entwistle et al. 2019 and references therein). In some situations, ingestion is the main pathway (Ishtiaq et al. 2018). Mining operations are understood to have some of the highest concentrations of potential harmful contaminants derived through anthropogenic activities, along with the highest particulate emissions and the highest risk to both human and environmental health (Csavina et al. 2012b; Meyer et al. 2015).

Overall, erosion, flooding, deforestation and the contamination and consumption of ground and surface waters all act as stressors on health of local communities, depleting food supplies and delivering harmful elements into the food chain (Rajaee et al. 2015). Not good news.

## Insight 3—a public health doctor

The World Health Organisation has described health as a state of complete physical, mental and social well-being and not merely the absence of disease or infirmity (WHO 1946). Although this misses the spiritual elements of health, it remains the most quoted description. More enigmatic but more thought-provoking is health as “the strength to be human” (Fergusson 1993).

Moving from clinical medicine as a general practitioner, to become a public health physician, meant moving my focus from the patient before me, and the disease processes within them, to the community, and the processes that affect health at a population, rather than individual, level.

**Fig. 1:**
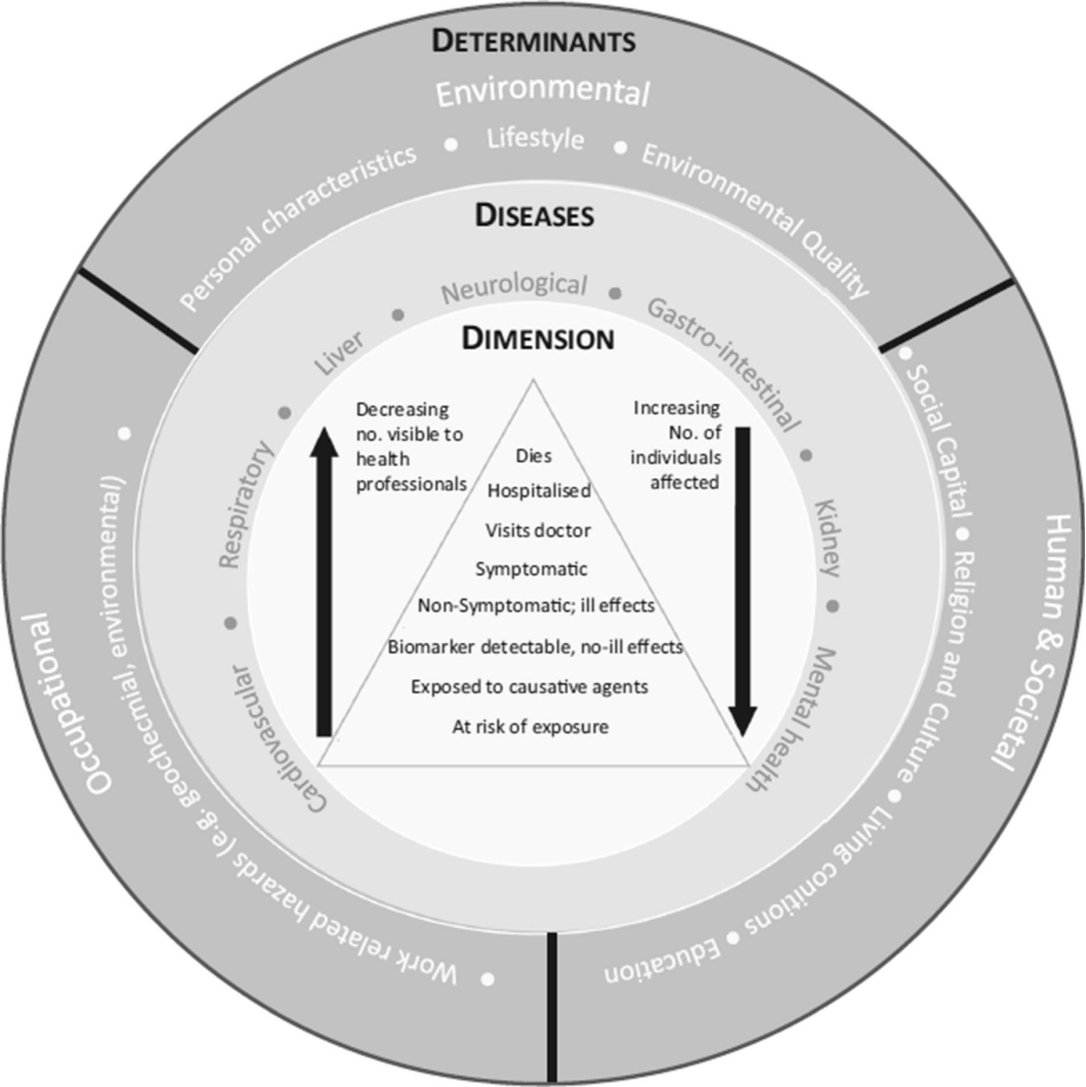
Mining-related determinants of disease, associated diseases and dimension (scale) of the disease burden [from Entwistle et al. (2019). Used freely under the Creative Commons Attribution 4.0 International License (http://creativecommons.org/licenses/by/4.0/); the ‘disease pyramid’© is after Stewart and Hursthouse (2018)]

The determinants of health are very important within public health (Dahlgren and Whitehead 1991); they are a diverse range of personal, social, occupational, environmental and economic factors which influence people’s mental and physical health; they may operate at the individual, community or international level and be environmental, employment-related or social (Fig. 1). Sometimes the term “wider determinants” is used to summarise the non-personal influences that mould health and illness. I noted that mining determinants are active in many different situations (Lewis et al. 2017) (Fig. 1). Not a simple issue.

### Environmental

Personal characteristics, such as age and sex, are important determinants, particularly in small-scale artisanal mining where children and women are more commonly involved than in large-scale industrial mining operations. Women and girls carry eggs of all their children so that any exposure to potentially harmful elements may affect the next generation as well as themselves (Sen et al. 2015; Appleton et al. 2017). Neurotoxic metals including arsenic, lead and mercury, as well as under- or over-exposure to essential trace elements such as zinc and manganese, are associated with perturbed foetal growth, adverse birth outcomes and cognitive and behavioural problems in later childhood (Sanders et al. 2015; Zheng et al. 2016). There is evidence that, although sperm is generated daily after puberty, there is transmission through sperm to the next generation of some early life exposures through epigenetic mechanisms; currently the evidence is limited to obesity, stress, risk of diabetic death, cardiovascular diseases and the like (Pembrey et al. 2014; Fernandez-Twinn et al. 2015), but it is possible that further work will add environmental metals to known epigenetic toxins such as endocrine disruptors (Marsit 2015).

Diet can be an important route of exposure to harmful contaminants, particularly when sourced from near the mines or from land affected by mining operations (Zhu et al. 2008). Nigeria has seen an outbreak of acute lead poisoning killing 400–500 children, mainly < 5 years of age, with thousands more affected, arising from the household processing of gold from artisanal mines contaminating food (Dooyema et al. 2012; Tirima et al. 2018).

The poor may not have a lot of choice in housing, often living close to mines or on top of mine waste (Demetriades 2011). House dust can be the main route of exposure of families, including children and pregnant women, who are usually the most vulnerable (Martin et al. 2014; Zota et al. 2016; Lewis et al. 2017).

The unrestricted use of metals leads to high levels of exposure. In particular, the use of elemental mercury in small-scale artisanal gold mining leads to methyl-mercury pollution of the local environment and ingestion through the locally grown diet. Inhalation of elemental Hg by the children and other workers, often working in kitchens away from the actual mine, is an issue (Basu et al. 2015). Mercury is toxic to the brain (mad hatter’s disease) and growing nervous system (Minamata disease).

### Occupational

Employment issues are important, different occupations having different risks and exposures. For example, in Turkish open-cast mines, surface installations, workshops and the mining area itself have the highest probability of serious, non-fatal accidents, which occur mainly in transport and manual handling (Onder and Mutlu 2016). Ergonomic hazards are usually minimised in large-scale mining, due to the highly mechanised state, but are constantly present in small-scale mining. Back pain, upper limb pain, lower limb pain are common (Jiménez-Forero et al. 2015). Fractures and contusions are the most frequently occurring injuries in small-scale mining, with collapse of the mine pits, drowning, crushing and falls the most frequently reported cause of accidents (Kyeremateng-Amoah and Clarke 2015; Basu et al. 2015). Overall, the rates of small-scale mining injuries in Ghana far outstrip the rates in the large-scale mines of South Africa or the USA. Safety knowledge is very limited (Calys-Tagoe et al. 2015; Long et al. 2015).

Safety aspects in work (notably role conflict, role ambiguity, quantitative job insecurity, or managerial issues) and of coping (namely avoidance style or changes in the work situation) contribute to compliance (or not) with safety procedures (Wysokiński et al. 2015; Zhang et al. 2016; Jacobs and Pienaar 2017) and the potential for injury from accidents. Migrant miners returning home to Botswana from South Africa have missed surveillance, resulting in not being diagnosed or compensated for occupational disease (Steen et al. 1997). Unfortunately, the situation affects South African miners as well as migrants and has only slowly improved in the intervening years (Kistnasamy et al. 2018).

Recycling of Waste Electrical and Electronic Equipment (WEEE) is increasing, but exposes the recycling workers to complex contaminant mixtures of metalliferous dusts associated with precious metals and rare earth elements, mixed with the organics and plastics found in such equipment that can adversely affect health (Lau et al. 2014; Annamalai 2015; Cesaro et al. 2018), so needs the same considerations as mining in terms of surveillance and control.

### Human and social

Migration to find work in mines often results in poor infrastructure and crowded living conditions which, allied with lack of social cohesion and support increases the risk of pathogen exposure (e.g. HIV, tuberculosis) and stress (Basu et al. 2015). South African miners have the highest incidence of tuberculosis (2500–3000 cases per 100,000 people) of any working population in the world (World Bank 2014). Across southern Africa, the triple disease burden of silicosis, HIV infection and tuberculosis among the very large population of miners and ex-miners constitutes a public health disaster: the overall mortality rate in ex-miners is 20% higher than that of the general population; white ex-miners had a 62% excess mortality relative to the white general population; ex-miners aged 20–24.9 years had a 79% higher mortality rate than the general population of the same age. There was also a hint of significant administrative underestimation of deaths, while miners working in exclusively gold mines had a greater mortality rate than those working in exclusively platinum mines; both groups had a far greater mortality rate than miners in exclusive coal mines. (Bloch et al. 2018). Mining is unhealthy, whether you come from a healthy population (white or younger than 25 years) or one with underlying higher rates of ill health (black community). The combination of silicosis and HIV infection is known to be a potent risk factor for incident tuberculosis among gold miners (Corbett et al. 2000), while miners with silicosis have been shown to have higher mortality rates while on tuberculosis treatment than miners without silicosis (Churchyard et al. 2000).

Psychological distress levels arising from poor social networks have been noted to contribute to raised levels of mental stress and mental health issues in miners (Considine et al. 2017). Chronic stress is a factor in a number of diseases, including anxiety, depression, sleep loss, all leading to poor memory and decision making; it impairs the immune system, increasing susceptibility to infections; it increases blood pressure, heart rate, cholesterol and triglycerides, contributing to heart disease, atherosclerosis, stroke, obesity and diabetes mellitus. On the contrary, good social support is protective (Mariotti 2015).

Living conditions have major influences on health. Poverty leads many into small-scale mining and holds them there, in a vicious cycle of limited investment funding leading to dependence on foreign equipment, low productivity and sponsor dependence, which in turn lead to low earnings, exacerbating poverty and unemployment with resulting limited investment funding. And so the cycle continues (Wilson et al. 2015).

War also has significant effects on health. As already noted, in the Democratic Republic of Congo militia groups control tantalum mining and trade by brutality, murder and rape (Bell 2014a, b). Diamonds have fuelled conflict in Angola, the Democratic Republic of Congo, Ivory Coast, Liberia and Sierra Leone. Countries rich in natural resources such as cobalt, coltan, cassiterite, copper and gold are often marred by corruption, authoritarian repression, militarisation and civil war. Rebel groups, governments and mining companies exploit mineral resources, fuelling civil and interstate conflict over control over riches. The local population suffers deprivation, control of food supplies and transport, physical and emotional abuse, as well as social destruction and displacement, with increases in a wide range of diseases, all exacerbated by reduced health care (see Global Policy Forum 2005–2019 and reports therein; Sidel and Levy 2008).

Perhaps the greatest pressure on mining is the current economic model that demands profit, seemingly at all costs, from any and every business, large or small. This distorts the three pillars of sustainable development, namely the environment, social equity and economic development (Jarvis et al. 2016). The perceived need to enhance shareholders’ return on their investment distorts these equal pillars, with profitability being the “bottom line”, the goal, almost at all costs. Miners, like many other employees in the vast majority of commercial enterprises, suffer as a result.

For example, fatigue is an increasingly relevant concern owing to extended shifts and overtime, driven by this distorted economic model. The implementation of fatigue risk management programs is growing within the mining industry (Wesdock and Arnold 2014), a positive result but one that treats the symptoms, not the cause. The International Agency for Research on Cancer has raised concerns about disruption of circadian rhythms through shift work (IARC 2010), again, an issue of the symptoms of the distorted economics that put profit above people.

Just over 1 billion people (~ 15% of the world’s population) live in extreme poverty. Most of them are in low- and middle-income countries; economic growth is seen as the clearest path to improving their lot. The size of the necessary growth to achieve this has significant consequences, one of which is an exponential increase in mineral consumption (Rogich and Matos 2008). And the need for rare elements for the ubiquitous electronic gadgets of the modern world means that we are all involved in the pressures to increase and improve mining, pressures that continue to disadvantage miners and the poor. Not an encouraging insight.

In Peru, increases in mining activity has been accompanied by fear in local, largely poor, communities that mining projects will contaminate their essential land and water resources. But mining is a significant economic resource in Peru and, as a result, the government has criminalised social protests. Also, some mining and oil extraction companies have exacerbated social stress and tensions by using private security forces, some of which have been accused of violating human rights (Slack 2009). Not a good outcome.

### Multiple determinants

Few diseases are unifactorial. Pneumoconioses (plural) are a group of dust-induced lung diseases and include coal miner’s pneumoconiosis (black lung disease), asbestosis and silicosis (Mandrioli et al. 2018). The incidence of black lung disease amongst coal miners in the USA and Australia has been dropping for a number of years but has recently begun to increase again (Graber et al. 2017; Laney et al. 2017).

The development of this severe, preventable, but incurable lung disease among miners depends on the type and grade (rank) of coal being mined, type or site of coal mine, chemical composition of dust, fineness of dust, concentration of dust in the air, length of period of exposure and underlying health status of the exposed worker, possibly including specific genes (Gamble et al. 2012; Blackley et al. 2014; Han et al. 2015; Liu et al. 2017; Perret et al. 2017). Smoking increases the risk of the disease (Altinsoy et al. 2016; He et al. 2017), while tuberculosis is commoner in miners with a pneumoconiosis (Leung et al. 2012; Mo et al. 2013; Ngosa and Naidoo 2016).

However, the recent increase in the incidence of coal miner’s pneumoconiosis is likely related to a lack of regulation of engineering dust controls in mines, even in high-income countries, despite this being the primary preventative measure, along with good surveillance of the miners (Perret et al. 2017). While it is possible that surveillance has improved, thus finding more cases, it is more likely that the dust control is not as good as it should be due to economic drivers for sustaining or increasing profit margins. Certainly, exposure surveillance schemes that rely on industry to police itself alone have often failed owing to economic pressures (Cohen et al. 2016).

The current definition of silicosis was adopted internationally in 1930. It gave a major improvement in the recognition of occupational pneumoconioses, but limited the pathogenic effect of silica to pneumoconiosis. This has led to the under-identification of other adverse health outcomes of silica and was due to economic pressures to reduce compensation payments (Rosental 2015).

### Interventions

Public health advocates prevention and remediation. Work characteristics associated with psychological distress (such as alcohol use, work role and satisfaction, financial factors and job security), vibration, specific occupational disorders (such as musculoskeletal, respiratory and auditory disorders), injury in artisanal mining, as well as dust generation (as noted above, the primary preventive measure), are modifiable (Long et al. 2015; Jiménez-Forero et al. 2015; Burström et al. 2016; Considine et al. 2017; Perret et al. 2017).

Calls for further action continue to be made throughout the literature, for specific health risks, with specific approaches, in specific countries (see, for example, Hermanus 2007; Taylor et al. 2014; Singh et al. 2014; Kyeremateng-Amoah and Clarke 2015; Basu et al. 2015; Jiménez-Forero et al. 2015; Utembe et al. 2015; Zhang et al. 2016; Haas and Mattson 2016; Considine et al. 2017). The case for surveillance, including biomonitoring, to support improved enforcement of health and safety legislation in order to protect both workers and the wider community against the hazards posed by mining activities, is also clear (Nemery et al. 2018).

Control measures for multi-factorial risks need to be multi-disciplinary (Mahoney et al. 2015). In the Nigerian lead outbreak, control measures included environmental remediation, chelation therapy, public health education and control of mining activities (Dooyema et al. 2012). Involvement of miners in the development and deployment of control measures is vital.

Successes have been recorded. For example, noise from earth-moving equipment, blasting, drilling and crushing can have a number of physical effects on health, including raised blood pressure, but noise exposure has been reduced (US mines: Roberts et al. 2017). Perhaps the biggest success has been in the reduction in the death rate from accidents in the US coal mining industry, from thousands per year 100 years ago (> 300 deaths per 100,000 miners, 1901–1910) to under 20 per year 2011–2018 (14/100,000) (derived from 1900 to 2018 data in MSHA n.d.). It shows what can be achieved.

Innovative approaches to improve safety and health are being considered. The development of end-of-shift self-assessment tools has the potential to improve engineering monitoring and give better evaluation of control technologies (Cauda et al. 2016), at least in high-income countries. Improvements in artisanal miners’ health are more difficult. Quite a challenge.

## Insight 4—a volunteer

But I am now retired, escaping from the “rat race” and able to volunteer to support good causes or just do something I enjoy at my own pace. I work on an occasional basis alongside employed sailors to offer a boat service on the nearby lake. I work for free; they work for a wage. It has given me time to consider who pays for miners’ ill health, their exploitation in many (but hopefully not all) situations.

I have recently visited the town of Elliot Lake in Ontario for personal reasons. Elliot Lake was “the uranium capital of the world” in the period from the mid-1950s to the late 1970s (Elliot Lake 2018). While the greatly increased mortality from radon-related lung cancer, probably enhanced by arsenic exposure in the gold mining areas, in uranium miners across Ontario has been reported (Kusiak et al. 1993), there has been no investigation reported of whether or not there is a risk to the local human communities from mine tailings and the use of mine waste in building materials, although the risk from radon to the ruffed grouse has been assessed around Elliot Lake (Clulow et al. 1992). The drinking water content of radon (^226^Ra) in the town remains below the Canadian maximum acceptable concentration (Chen et al. 2018), although the health risk from radon is gaseous, and so soil and rock also need assessing, as does ingress into houses. Indeed, although both the city museum and the local monument to the miners recognise industrial accidents and illness, that is all. No detail, no specifics, no explanation, no deliberation. A similar picture emerges of the many centuries of mining for a wide variety of minerals and elements in the English Lake District, where I now live (Lake District National Park n.d.; Visit Cumbria n.d.). Not a good reflection on health or government leadership.

However, mining brings employment, improves some infrastructure and sometimes health care (Mining Health Initiative 2013), possibly education.

But the employment does not always lift people out of the poverty trap (Wilson et al. 2015); indeed, employment, even self-employment, is insecure for many (Ssekika 2017). Health services for miners and their communities, run in partnerships by government and mining companies, tend to produce mixed results in terms of the health of the community (Stephens and Ahern 2001) since they are not addressing the wider causes of the ill health, including economics and the poverty trap. And as for education? How can this improve the lot of child labourers, whose day is spent labouring, supporting both their parents and the mines and therefore unable to attend school (Segawa 2016)?

I conclude that we all pay for mining. As John Donne (1572–1631) wrote (*For Whom the Bell Tolls*), No man is an island/Entire of itself.

## Insight 5—a lay preacher

The Wellcome Trust and Gates Foundation have been chastised for, on the one hand, funding health services while, on the other, investing in fossil fuel companies whose mining operations may have a profound and adverse effect on the local communities (Smith and Carrington 2015). I am interested in practical issues around ethics and the reason people make ethical decisions. Our modern way of life cannot do without mining, but—is ethical mining possible or do we accept as inevitable the cost of miners’ poor health? Can ethical mining improve miners’ health? Will it lead to decreased profits?

Exploitation takes place outside the realms of international agreements such as the Rio Declaration on Environment and Development (http://www.un.org/documents/ga/conf151/aconf15126-1annex1.htm). Ultimately the polluter is the purchaser of goods and services at a rate which does not include fair wages and clean up (or prevention) of pollution. We all need to act.

The reconciliation of the varied, and sometimes competing, interests of the individual, society, business and the state through individual choice, government responsibility, business profit and corporate social responsibility is fraught with difficulties (Krebs 2008; Benton 2018). There are many underlying ethical theories: Aristotle’s virtue ethics focuses on the moral character of the agent, Immanuel Kant’s on doing right from a sense of duty, John Stuart Mill’s considers the balance of harm and benefit to the individual and society from actions (Ortmann et al. 2016), and there are several others. Ethical approaches that are not underpinned by explicit theory can be rightly criticised (Kar-Purkayastha 2009). As a Christian lay preacher my own ethics are based on the words of Jesus, “Love God, love your neighbour as yourself” (Bible, Matthew 22:39). Not an approach without challenges. Or possibilities. This paper is one of my responses.

But mining companies can improve health (Davey 2018). For example, corporate social responsibility (Meier et al. 2014) in Zambian copper and nickel mining, encouraged by the financial backers, has resulted in improved health in children and women living near the mines, although, yet again, the poor have disproportionally lost out (Knoblauch et al. 2017, 2018).

## Conclusions

As noted nearly 20 years ago, “Mining remains one of the most hazardous occupations in the world…” (Stephens and Ahern 2001). Donoghue wrote (2004), “… although substantial progress has been made …, there remains room for further risk reduction…”, words that are as true now as then. Continuing increased reductions in risk and exposure that would improve the health of miners in a sustainable way to benefit the miners, their families and the rest of the world are still urgently needed and are touched on in many papers (e.g. Basu et al. 2015; Jiménez-Forero et al. 2015; Kyeremateng-Amoah and Clarke 2015; Long et al. 2015; Liao et al. 2016; Considine et al. 2017; Perret et al. 2017).

But academics need to work in partnership: with communities, government and industry, to develop multi-disciplinary, evidence-based solutions to the issues around mining resources, health and sustainable development (Maier et al. 2014). Employment, health, economic stability and environmental protection need not be mutually exclusive (Woodward and Hales 2014).

We all have a part to play in that.
